# Guanidine Hydrochloride-Induced Hepatitis B Virus Capsid Disassembly Hysteresis

**DOI:** 10.1021/acs.biochem.4c00077

**Published:** 2024-05-24

**Authors:** Daniel Khaykelson, Roi Asor, Zhongchao Zhao, Christopher John Schlicksup, Adam Zlotnick, Uri Raviv

**Affiliations:** † Institute of Chemistry and the Center for Nanoscience and Nanotechnology, the Hebrew University of Jerusalem, Edmond J. Safra Campus, Givat Ram, Jerusalem 9190401, Israel; ‡ Department of Molecular and Cellular Biochemistry, Indiana University, Bloomington, Indiana 47405, United States

## Abstract

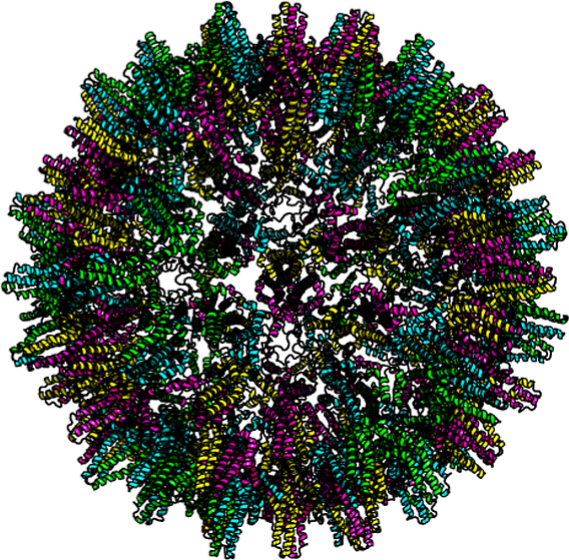

Hepatitis B virus (HBV) displays remarkable self-assembly capabilities that interest the scientific community and biotechnological industries as HBV is leading to an annual mortality of up to 1 million people worldwide (especially in Africa and Southeast Asia). When the ionic strength is increased, hepatitis B virus-like particles (VLPs) can assemble from dimers of the first 149 residues of the HBV capsid protein core assembly domain (Cp149). Using solution small-angle X-ray scattering, we investigated the disassembly of the VLPs by titrating guanidine hydrochloride (GuHCl). Measurements were performed with and without 1 M NaCl, added either before or after titrating GuHCl. Fitting the scattering curves to a linear combination of atomic models of Cp149 dimer (the subunit) and *T* = 3 and *T* = 4 icosahedral capsids revealed the mass fraction of the dimer in each structure in all the titration points. Based on the mass fractions, the variation in the dimer–dimer association standard free energy was calculated as a function of added GuHCl, showing a linear relation between the interaction strength and GuHCl concentration. Using the data, we estimated the energy barriers for assembly and disassembly and the critical nucleus size for all of the assembly reactions. Extrapolating the standard free energy to [GuHCl] = 0 showed an evident hysteresis in the assembly process, manifested by differences in the dimer–dimer association standard free energy obtained for the disassembly reactions compared with the equivalent assembly reactions. Similar hysteresis was observed in the energy barriers for assembly and disassembly and the critical nucleus size. The results suggest that above 1.5 M, GuHCl disassembled the capsids by attaching to the protein and adding steric repulsion, thereby weakening the hydrophobic attraction.

## Introduction

About half of the known viral capsids are icosahedral.^1^ Capsids must have a narrow stability window to both protect and release their genome for successful infection to occur. Hence capsids self-assemble via weak noncovalent interactions.^2^ Hepatitis B virus (HBV) is an enveloped dsDNA virus where the capsid serves as a metabolic compartment for the synthesis of DNA from the RNA pregenome and a vehicle for delivering the genome to the nucleus (for infection) or the cell membrane (to generate new infectious particles), a partially double-stranded DNA-enveloped virus. In vivo and in vitro, the capsid protein (Cp) self-assembles into 120-homodimer *T* = 4 capsids (95%) and 90-mer *T* = 3 capsids.^3^ In vivo, about 90% of the assembled capsids are empty and self-assembled with high fidelity in the crowded environment of the cell from a self-assembling homodimer.^4^

The structure of the Cp149 dimer encodes two interactions controlling the assembly: electrostatic repulsion and hydrophobic attraction.^3,5−7^ Therefore, increasing ionic strength and/or temperature can trigger in vitro capsid assembly.^8^ However, rapidly increasing the ionic strength too much can lead to kinetically trapped capsids.^9,10^ In vitro virus capsid disassembly can be triggered by changes in pH,^11,12^ applying mechanical forces,^13,14^ heating,^14,15^ or addition of chaotropes.^16^ These conditions provide insights into disassembly mechanisms and the forces regulating and stabilizing the capsid.^17^ Whereas changes in pH may serve as a tool to probe the electrostatic character of Cp149 dimers and their interactions,^11,18−20^ the addition of chaotropes such as guanidine hydrochloride (GuHCl) may serve as a useful tool to inspect the hydrophobic interactions between dimer subunits. GuHCl is used as a common denaturing agent in various biochemical systems^21,22^ and avoids the interaction with water molecules on its planar surface,^23^ as it does when adsorbing on hydrophobic surfaces.^21,24^

In this work, we used solution small-angle X-ray scattering (SAXS) to resolve the effect of GuHCl titration on the disassembly of empty capsids assembled from Cp149. SAXS has the temporal and spatial resolutions to study the structure and dynamics of viruses in solution.^25−27^ To extract thermodynamic properties such as the dimer–dimer self-association standard free energies, we used the advanced SAXS data analysis tools recently developed in our lab.^9,10,28^

GuHCl titration experiments were performed with and without 1 M NaCl, added either before or after the GuHCl titration. Ionic strength stabilized the capsid. Therefore, high ionic strength was used to create capsids at steady state from which disassembly reactions commenced. A significant fraction of capsids disassembled at high GuHCl concentrations. The SAXS data were analyzed by fitting the scattering curves to a linear combination of the three most dominant structures under a wide range of assembly conditions,^9,10^ including a Cp149 dimer, *T* = 3 capsid, and *T* = 4 capsid. The dimer–dimer self-association standard free energy as a function of GuHCl concentration was determined based on the mass fraction of the dimer in each structure. Using these data, we estimated the assembly energy barrier, the critical nucleus size, and the disassembly energy barrier. Extrapolation of the standard free energy to [GuHCl] = 0 revealed disassembly hysteresis compared with the corresponding assembly reaction. In other words, the dimer–dimer association and dissociation free energies differed significantly. The results suggest that although the added GuHCl increased the ionic strength (which strengthened the association free energy), it weakened the hydrophobic interactions by either attaching to the dimer–dimer interface or enhancing the dimer solubility.

## Materials and Methods

### Capsid Protein

N-terminally truncated HBV dimer, Cp149, was expressed in E. coli using a pET 11-based vector and purified as previously described.^29^ To remove any aggregates before experiments, the dimer was incubated in 3 M urea for 1.5 h at 4 °C. A buffer exchange to 50 mM HEPES, at pH 7.5, was then applied using a PD10 column at 4 °C.^9,10^

### Assembly and Disassembly Experiments

Three types of assembly experiments were performed.

1 2 mg/mL (56 μM) Cp149 was first assembled in 200 mM NaCl for 5 h, followed by an overnight (∼11 h) incubation at 36 °C, then a dialysis against 0.5 M NaCl for 2.5 h, and finally a dialysis against 1 M NaCl for 4 h. Following this protocol, the total protein concentration decreased to 1.42 mg/mL. The assembled capsid solution was then mixed, at a 1:1 volume ratio, with solutions containing 1 M NaCl and increasing concentrations of GuHCl. The final capsid protein concentration was 0.71 mg/mL (20 μM).
2 1.4 mg/mL (40 μM) Cp149 was first assembled in increasing concentrations of GuHCl and then NaCl was added to get a final concentration of 1 M.
3 1.4 mg/mL (40 μM) Cp149 was assembled in increasing concentrations of GuHCl.

### SAXS Measurements

We measured the SAXS data at the ID02 (headed by T. Narayanan)^30^ and BM29 (headed by P. Pernot)^31^ beamlines in the European Synchrotron Radiation Facility (ESRF), Grenoble. A detailed description of the experimental setup was presented elsewhere.^9,10^ Measurements of water were used for calibration of the absolute intensity.^9^ Before and after each sample, we measured the buffer background under conditions identical with those of the samples. Scattering 2D patterns were normalized to the intensity of the transmitted beam and azimuthally integrated to yield the scattering intensity curve, *I*, as a function of the magnitude of the scattering vector, *q*.^32^ Background buffer scattering curves were averaged and subtracted from its sample intensity curve to give the final background-subtracted scattering intensity curves of the assembly reactions, as explained.^33−38^

### Modeling the Scattering Curves

**Figure 1:**
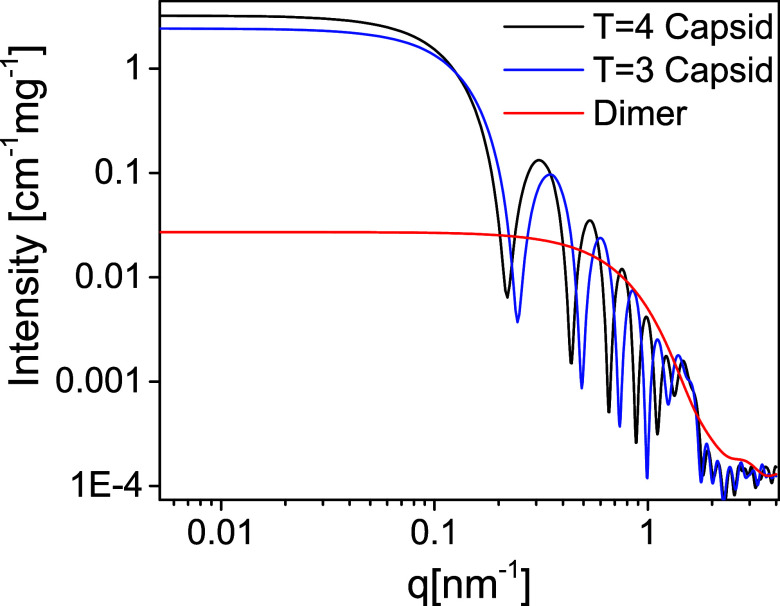
Computed scattering curves from atomic models of Cp149 dimer, *T* = 4 capsid, and *T* = 3 capsid. Scattering intensity is plotted as a function of the magnitude, *q*, of the scattering vector. These computed spectra were used to fit the experimental data and are identical to the models used in our earlier publications.^9,10^.

The models used to fit the data were previously described.^9,10^ Briefly, a library of representative possible *T*_*n*_^s,c^ intermediates, varying from a dimer to full capsid, were generated and used without further modifications.^9,10^*T*_*n*_^s,c^ is an icosahedral capsid intermediate, whose triangulation number is *n*, and is made of s capsid protein dimer subunits, held together by c dimer–dimer contacts. The most compact models were those with a maximum number of contacts (i.e., most stable) and were therefore selected for the fit (Figure 1).

The scattering amplitude of the solvated dimer was calculated^9,10^ using the atomic coordinates of Cp149 dimer (PDB ID 2G33). The electron density of the solvent was set to the electron density of water (334 *e*/*nm*^3^) and modified based on the concentration of GuHCl (see below).^22^ The thickness and electron density of the solvation layer around the dimer atomic model were 2 Å and 363 ^e^/_nm_^3^, respectively. These parameters were found to best fit the scattering data from a solution of Cp149 dimers in our earlier studies.^9,10^ The scattering amplitude of the solvated dimer was then used for calculating the scattering intensity, , of all the other intermediates, according to
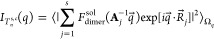
where  is the scattering amplitude of the *j*th solvated dimer whose orientations in the complex *T*_*n*_^s,c^ are given by the rotation matrices **A**_***j***_, in the Tait–Bryan convention.^28^ is the geometric center of the *j*th dimer, and ⟨···⟩_Ω*q*_ represents the orientation averaging of the scattering intensity in the reciprocal space.

To account for electron density contrast at different GuHCl concentrations, the electron density of each concentration was calculated.^22^ The electron densities of 1.5, 2, 2.3, and 2.6 M were 344, 347, 349, and 351 ^e^/_nm_^3^, respectively. These electron densities were used to compute the  models in D^+^.^28^ Up to *q* = 3 nm^–1^, the differences compared with water (electron density of 334 ^e^/_nm_^3^) were only a scaling factor, with the largest scale difference being 1.7 for *T* = 4 at 2.6 M GuHCl. At each GuHCl concentration, the scaling factors of the models were similar. The largest scaling factor difference was 0.1 and occurred between the dimer and *T* = 4 capsid. Hence, at each GuHCl concentration, the relative differences in the mass fraction of Cp149 in each of the models were insensitive to the differences in the electron densities of the solvent.

### Dimer–Dimer Self-Association Standard Free Energy

Calculations of the dimer–dimer association standard Gibbs free energy per contact in a capsid, Δ*G*_cc_^◦^, in the molar concentration scale are explained in our earlier studies.^9,10,16^ Briefly, the equilibrium constant of an HBV capsid containing *s* dimers of Cp149 is
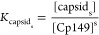
1and
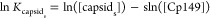
2The capsid standard Gibbs free energy in the concentration scale is then
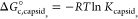
3where *R* is the gas constant and *T* is the absolute temperature. The standard Gibbs free energy per contact, Δ*G*_cc_^◦^ in the concentration scale, is obtained by dividing the total capsid standard Gibbs free energy, , by the total number of dimer–dimer contacts, c (240 or 180 for complete *T* = 4 or *T* = 3 particles, respectively).

The molar fraction of a capsid, containing *s* dimers of Cp149, is *X*_s_/s where *X*_s_ is the molar fraction of Cp149 dimers in capsids of size s. *X*_s_ is obtained from the initial concentration of Cp149 protein (where 1 mg/mL Cp149 = 28.2 μM). Taking the water concentration as the total number of moles (55.5 M)
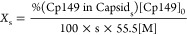
4where [Cp149]_0_ is the initial added protein concentration and %(Cp149 in Capsid_s_) is the percentage of the contribution of the model of capsid_s_ to the fit of the scattering curve.

Using the molar fractions of the dimer, *X*_Cp149_, and capsid of size s, *X*_capsids_, the total capsid standard Gibbs free energy in the molar fraction scale becomes
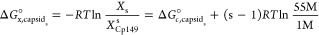
5where the last term is needed to switch between the molar concentration scale  and the molar fraction scale of the standard free energy , as explained.^10^ Dividing  by the number of contacts, *c*, gives the Gibbs standard free energy per contact, Δ*G*_*cx*_^◦^, in the molar fraction scale.

### Estimating the Energy Barrier and Critical Nucleus Size

Following earlier nucleation and growth analysis,^39−42^ the energy barrier for the assembly of a capsid with a triangulation number T containing *s* subunits is
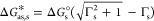
6and the critical nucleus size is
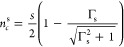
7where Δ*G*_s_^◦^ is the maximum barrier height, given by sα_s_/2, and Γ_s_ ≡ – Δμ_s_^◦^/α_s_ is the dimensionless measure of the supersaturation, driving the assembly of a capsid with a triangulation number *T* containing *s* subunits. The change in the standard chemical potential of a capsid protein in the assembled and the dissociated states is Δμ_s_^°^ = −ln(*X*_Cp149_^Tot^/*X*_Cp149_^*^) in units of thermal energy where *X*_Cp149_^*^ is the critical Cp149 dimer molar fraction beyond which capsid starts to assemble and *X*_Cp149_^Tot^ is the total capsid protein molar fraction. α_s_ = 4π*R*_s_σ_s_/*s* is a dimensionless magnitude of the rim energy, where *R*_s_ is the capsid radius. σ_s_ is the free-energy cost per unit length of the rim, estimated by σ_s_ = −*cg*_s_/*r*_s_, where *c* is the fraction of bonds that a rim protein has compared to a core protein, estimated to be ∼0.3. *r*_s_ is the effective diameter of a protein subunit, approximated as a disk and estimated as ,^43^ hence . The HBV capsid radii are *R*_90_ = 14 nm (*T* = 3) and *R*_120_ = 16.5 nm (*T* = 4).^9^ Using these data, we get *r*_s_ ∼ 6 nm.  is the average interaction energy per capsid protein subunit in units of thermal energy in a capsid with a triangulation number *T*, averaged over all the *s* subunits making the fully formed capsid. Hence, 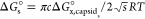 and 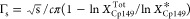


## Results and Discussion

In our earlier papers, we showed that HBV capsid assembly reactions are entropically driven and attain equilibrium under moderate assembly conditions, in which the interaction between subunits is weak.^8−10,44−46^ Under those conditions, most subunits are found in either dimer or the complete capsid form.^2,9,10,47,48^ Capsid disassembly requires the removal of dimers from the capsid structure and therefore overcomes the dimer–dimer association free-energy barrier. Four dimer–dimer contacts should be simultaneously dissociated to remove the first dimer from a complete capsid.^16^ The dimer–dimer association free energy decreases (i.e., becomes more negative) with ionic strength,^9,10^ suggesting that disassembly should become less favorable with increasing ionic strength, as was shown for urea-triggered disassembly.^16^

All-atom molecular dynamic simulations revealed that the capsid of HBV is extensively breathing.^49,50^ These results are supported by biochemical observations.^51^ The sensitivity of mature capsids to spontaneous dissociation^52^ suggests that nonenveloped capsids have a finite time before they spontaneously disassemble. Intracellular crowding likely has a stabilizing effect because it will increase the local concentration of capsid protein, which has been shown to promote capsid assembly.^10^ Denaturants can emulate the effect of internal pressure.

We examined capsid disassembly by the chaotrope GuHCl,^53^ under three limiting conditions, selected to affect capsid stability without unfolding Cp149 homodimers.^9,10^ In the first limiting case, we examined the effect of GuHCl on free Cp149 dimers in solution. As GuHCl solubilizes the hydrophobic residues of proteins, it may have preferential interactions with the exposed hydrophobic regions of the dimers in the solution. Buried hydrophobic residues may interact with GuHCl and increase the solubility of the dimer.

**Figure 2:**
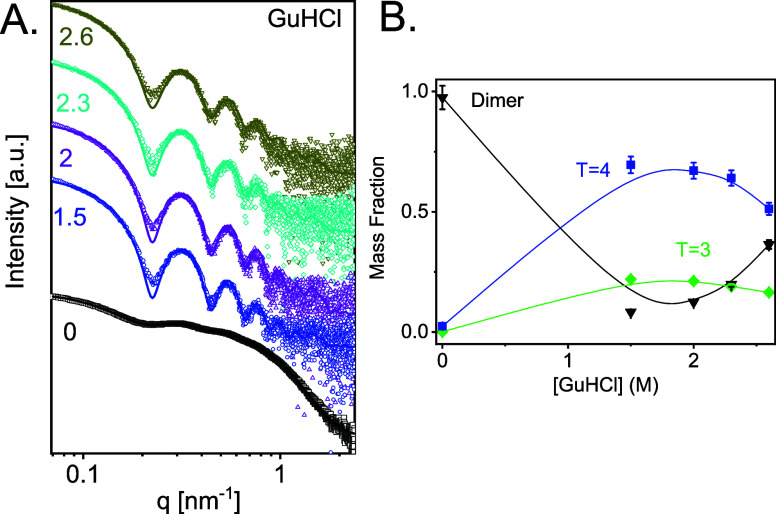
Effect of GuHCl on HBV capsid assembly. (A) SAXS data (open symbols) from 42 μM Cp149 dimer, incubated at 36 °C with different GuHCl concentrations, as indicated (in molar units). The scattering curves were fitted to a weighted linear combination of the computed scattering curves from atomic models of dimer, *T* = 3 capsid, and *T* = 4 capsid (solid curves). (B) Mass fraction of Cp149 dimer, *T* = 3 capsid, and *T* = 4 capsid as a function of GuHCl concentration, used for fitting the models in A. SAXS curves were measured at the ID02 beamlines at the ESRF.

We measured the SAXS curves from solutions of 1.4 mg/mL (42 μM) Cp149 dimers in 50 mM HEPES in increasing concentrations of GuHCl (Figure 2A). We note that this protein concentration is needed to obtain a good SAXS signal-to-noise ratio but is much higher than that typically used for Cp149 assembly experiments. The SAXS data were fitted to a linear combination of three computed SAXS atomic models: a dimer, a *T* = 4 capsid, and a *T* = 3 capsid (Figure 1). The models could adequately fit the data (Figure 2A) and the analysis determined the mass fraction of each state as a function of GuHCl concentration (Figure 2B). Additional intermediate structures did not significantly improve the fit. The bulk electron density contrast in the GuHCl solutions was accounted for as explained in Materials and Methods. Based on repeated measurements, the resulting mass fractions of the dimer in each model were averaged and gave an error of about 5% in the mass fractions (Figure 2B). However, a two-state analysis (a dimer and a *T* = 4 capsid) was insufficient to explain the data, emphasizing the sensitivity of SAXS measurements to the presence of other species in the solution. Multiscale computational models of HBV assembly have supported a more granular investigation of HBV reaction product polymorphism including malformed structures.^54^ These suggest a structural basis for *T* = 3 and *T* = 4 capsids consistent with energetically favoring *T* = 4 symmetry. A similar SAXS data analysis was validated by comparing the mass fraction of dimer with size-exclusion chromatography analysis.^10^

We found that 1.5 M GuHCl induced the assembly of 42 μM Cp149 with a pseudocritical concentration of 3.3 μM. We attribute the assembly to ionic strength-dependent conformational change^55,56^ and the screening of the electrostatic repulsion between Cp149 dimers by the GuHCl ions.^57^ However, a further increase of the GuHCl concentration led to significant disassembly of mostly *T* = 4 particles (particularly at 2.6 M GuHCl) and assembly of *T* = 3 particles. As capsids still formed under those conditions, it is likely that 2.6 M GuHCl dissociates the capsid without denaturing the dimer, in agreement with an earlier study.^16^

It has been shown that GuHCl disrupts the interactions between large hydrophobic pairs.^58^ As GuHCl is weakly hydrated, it breaks fewer hydrogen bonds, while it accesses hydrophobic surfaces. In addition, its planar shape allows it to associate parallel to hydrophobic side chains, through van der Waals interactions.^59^ Hence, a hydrophobic association of GuHCl with the Cp149 hydrophobic interfaces could add a steric hindrance to the interaction between dimers, decrease the hydrophobic attraction (known to direct capsid formation), and inhibit assembly. Conversely, incubation in urea will likely lead to different results as urea has no contribution to the ionic strength.^21,24^

To examine the effect of GuHCl on the dimer–dimer interaction without the complication of assembly, we examined its interaction with capsid. Capsids were assembled through a stepwise dialysis protocol where the ionic strength was gradually increased to 1 M NaCl (see Materials and Methods). The initial assembly in mild conditions (200 mM NaCl) led to a small mass fraction of *T* = 3 particles.^9^ In addition, it allowed the assembly to follow a narrow path through compact and stable intermediates with minimal kinetic traps and malformed particles.^9,10^ Further dialysis against higher NaCl concentrations (0.5 and then 1 M) decreased the dimer–dimer standard free energy per contact, stabilized the capsids, and increased the barrier for disassembly.

**Figure 3:**
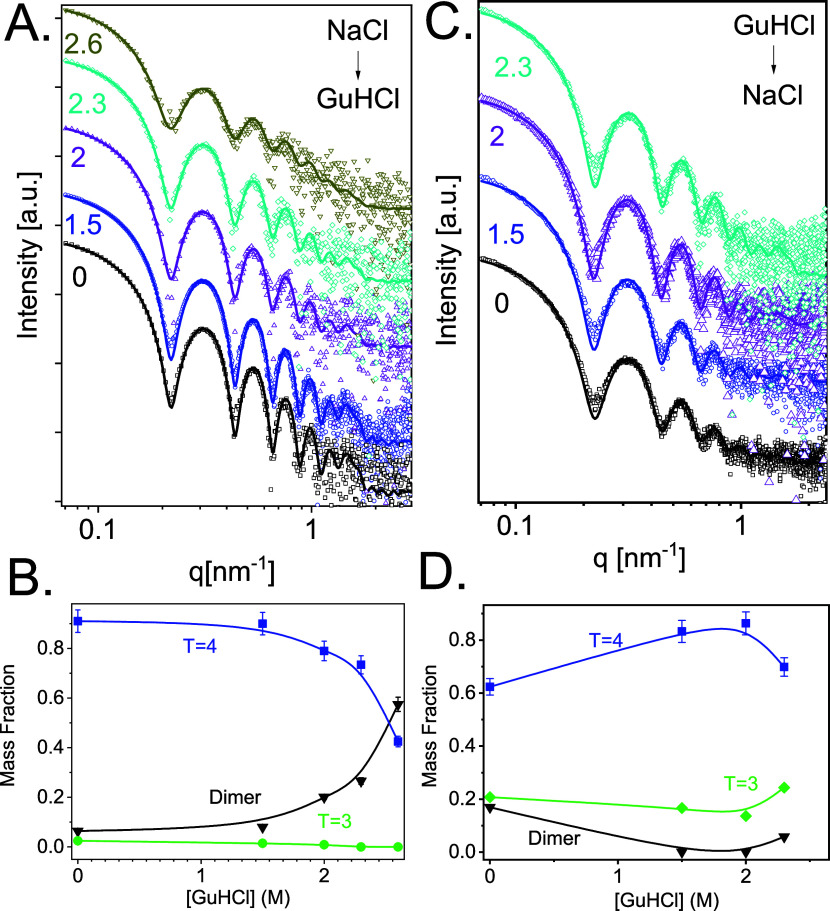
Effect of NaCl and GuHCl on HBV capsid assembly and disassembly. (A) Capsids were assembled from 1.42 mg/mL Cp149 in 200 mM NaCl and 50 mM HEPES, at pH 7.5. The capsids were then dialyzed against 0.5 and then 1 M NaCl (see Materials and Methods). The capsids were then mixed with a solution of 1 M NaCl and increasing concentrations of GuHCl at a 1:1 volume ratio. The mixed solutions were incubated for 24 h at 36 °C, after which SAXS curves were measured (open symbols) and analyzed as explained in Figure 2 (solid curves). (B) The resulting mass fractions of dimer, *T* = 3 capsid, and *T* = 4 capsid as a function of GuHCl concentration. (C) The experiments in Figure 2 were repeated and then 1 M NaCl was added in one step to each sample. SAXS curves were measured (open symbols) and analyzed as explained in Figure 2 (red curves). (D) The resulting mass fractions of dimer, *T* = 3 capsid, and *T* = 4 capsid as a funciton of GuHCl concentration. SAXS curves were measured at BM29 and ID02 beamlines at the ESRF.

We then examined the effect of titrating GuHCl on the assembled capsids with SAXS. The stabilized capsids were incubated in increasing concentrations of GuHCl and 1 M NaCl for more than 24 h. To characterize the disassembled structures, we fitted the SAXS data using the same three-state analysis (Figure 3A and B).

Capsids barely disassembled in the presence of 1.5 M GuHCl (Figure 3A,B). When the assembled capsids, however, were incubated at higher GuHCl concentrations, disassembly increased with the GuHCl concentration. The mass fraction of each model was calculated as a function of GuHCl concentration (Figure 3B and Table 1). The result in the absence of GuHCl is consistent with earlier analyses.^9,10^Figure 3B suggests that the relatively small fraction of *T* = 3 capsid disassembled before *T* = 4 capsid did.

**Table 1:** Mass Fraction of Dimer, *T* = 3 capsid, and *T* = 4 capsid, Obtained from Fits to the Disassembly Scattering Data (Figure 2)

GuHCl [M]	*T* = 4	*T* = 3	dimer
0	0.906	0.025	0.069
1.5	0.90	0.015	0.08
2	0.79	0	0.209
2.3	0.734	0	0.266
2.6	0.428	0	0.572

In the third set of conditions, the Cp149 dimer was first incubated in increasing GuHCl concentrations, then 1 M NaCl was added in one step, and SAXS measurements were performed and analyzed as explained (Figure 3C,). This set of conditions was designed to further test the competitive effects of ionic strength and solubilization of hydrophobic surfaces. The results in Figure 3D resembled the trend in Figure 2B. When 1 M NaCl was added in one step, however, the total fraction of capsids was significantly larger.

When 1 M NaCl was gradually added (Figure 3A,B), the fractions of dimer and *T* = 3 particles were lower, whereas the fraction of *T* = 4 was higher than when the NaCl was added in one step (Figure 3C,D). This difference can be understood by recalling our earlier SAXS and time-resolved SAXS results of the assembly reaction and the reaction energy landscape, determined based on the analysis of those experiments.^9,10^ These studies revealed that at low salt concentrations, capsid assembly follows a narrow minimum free-energy pathway, going through the most stable and compact intermediates with high energy barriers for kinetically trapped states. At high salt concentrations, however, the assembly barrier is low and kinetically trapped states accumulate. Gradual increase of the salt concentration considerably lowered the accumulation of kinetically trapped states, allowed optimal initial assembly of *T* = 4 particles at the lower salt concentrations, and reduced the concentration of free dimers. Upon increasing the salt concentration, the lower concentration of free dimers increased the energy barrier for assembly,^10^ and new assembly reactions followed narrow assembly pathways, similar to those of low salt concentrations, and avoided kinetically trapped states or off-pathway assemblies. Therefore, the reactions in which 1 M NaCl was added in a single step accumulated *T* = 3 particles, formed fewer *T* = 4 particles, and most likely did not fully attain equilibrium.

In the presence of 1.5 or 2 M GuHCl (Figure 3C and D), the fraction of *T* = 4 particles increased. However, when 2.3 M GuHCl was added, the fraction of *T* = 4 decreased, whereas the fractions of free dimer and *T* = 3 increased. When NaCl was gradually added before the GuHCl titration (Figure 3A,B), significant (14.5%) *T* = 4 disassembly was not observed until the GuHCl concentration was ≤2 M. When comparing with earlier data,^16^ performed at lower NaCl concentrations, it is clear that higher NaCl concentration resulted in more stable capsids; the midpoint in the mass fraction and all the curves were shifted to higher GuHCl concentrations by about 0.5 M.^16,28^

**Figure 4:**
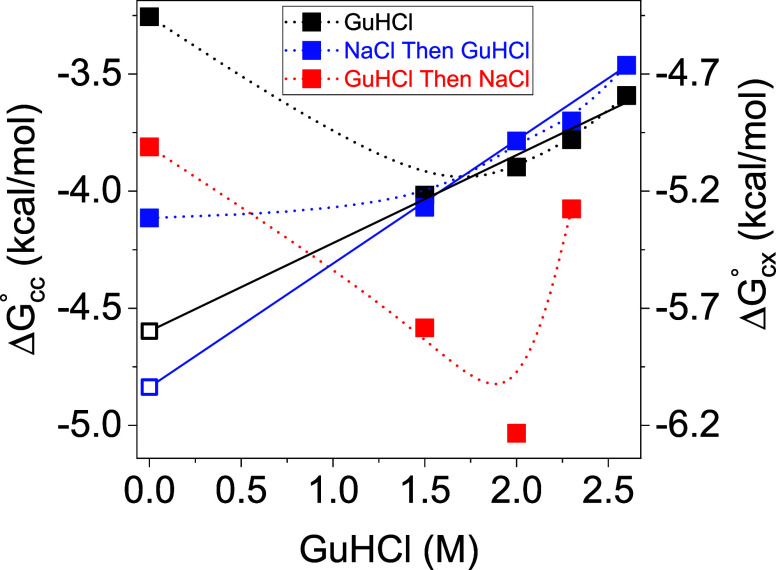
Disassembly hysteresis. The dimer–dimer association standard Gibbs free energy per contact in a *T* = 4 capsid on either the concentration, Δ*G*_cc_^◦^, or the molar fraction, Δ*G*_*cx*_^◦^, scales, as a function of GuHCl concentration when NaCl was not added (solid black symbols), when GuHCl was added after the concentration of NaCl was gradually increased to 1 M (solid blue symbols), and when GuHCl was first added and then 1 M NaCl was added in a single step (solid red symbols). Extrapolation to zero GuHCl concentration (solid lines) gives the extrapolated dimer–dimer disassembly standard free energy in 1 M NaCl (open blue symbols) and water (open black symbols), Δ*G*_cc_^°^(0 GuHCl)_d_ = −4.84 and −4.58 kcal/mol, respectively, on the concentration scale [or Δ*G*_*cx*_^°^(0 GuHCl)_d_ = −6.07 and −5.81 kcal/mol on the molar fraction scale]. These values are 0.75 and 1.4 kcal/mol lower than the observed dimer–dimer association Gibbs standard free energy in the absence of GuHCl,Δ*G*_cc_^°^(0 GuHCl)_a_, on the concetration scale [or Δ*G*_*cx*_^°^(0 GuHCl)_a_ on the molar fraction scale], in 1 M NaCl and in water, respectively. The free energies were calculated using eq 5. The dimer–dimer free energy for the assembly of *T* = 3 particles was slightly lower (99.4 ± 0.1%) than the *T* = 4 energies.

**Figure 5:**
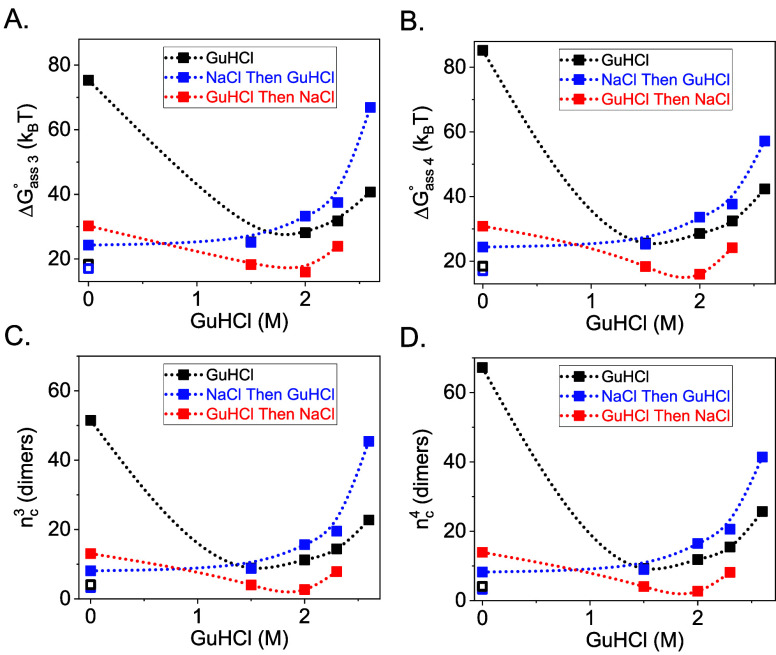
Estimated the assembly energy barriers, Δ*G*_as,s_^*^, in the molar fraction scale in units of thermal energy and the critical nucleus sizes *n*_*c*_^s^ (i.e., number of Cp149 dimers) of the different reactions, as indicated. The values were calculated based on eqs 6 and 7, using the mass fractions from Figures 2 and 3 (solid symbols) or the extrapolated dimer–dimer disassembly standard free energies in the absence of GuHCl, Δ*G*_*cx*_^°^(0 GuHCl)_d_, from Figure 4 and their associated mass fractions (see text), in water (open black symbols) or 1 M NaCl (open blue symbols).

The dimer–dimer association standard Gibbs free energy per contact on either the concentration, Δ*G*_cc_^◦^, or the molar fraction, Δ*G*_*cx*_^◦^, scales was calculated for all the experimental conditions (Figure 4), using the mass fractions of dimer, *T* = 3 capsid, and *T* = 4 capsid, and the number of contacts per capsid (240 for *T* = 4 or 180 for *T* = 3) as explained in the Dimer–Dimer Self-Association Standard Free Energy section. We have also used our data for estimating the energy barrier for assembly, Δ*G*_as,s_^*^, in the molar fraction scale and the critical nucleus size, *n*_*c*_^s^ (Figure 5), as explained in the Estimating the Energy Barrier and Critical Nucleus Size section. In the absence of GuHCl, a high assembly energy barrier and large critical nucleus size were observed in water, and both decreased when 1 M NaCl was added. The addition of up to 2 M GuHCl significantly decreased the assembly energy barrier and critical nucleus size in water, owing to the electrostatic screening effect of GuHCl. In the presence of 1 M NaCl, where the screening length was already short (∼0.3 nm), the initial addition of GuHCl had a much smaller effect. Further increasing the GuHCl increased the assembly energy barrier and critical nucleus size in all of the cases.

In our earlier study,^9^ our thermodynamic analysis of the SAXS data revealed slight variation in the association free energy per contact in the *T* = 4 and *T* = 3 symmetries. These small changes are magnified because the association energies are defined on a per dimer–dimer contact interaction, and capsids have 180 (*T* = 3) or 240 (*T* = 4) such contacts, resulting in a significant difference in the stability and concentration of the particles. In agreement with our earlier analysis,^9^ we found in this study that the standard association free energy per dimer–dimer contact in a *T* = 3 particle was only slightly below (99.4 ± 0.1%) the dimer–dimer contact in a *T* = 4 particle (Figure 4).

**Figure 6:**
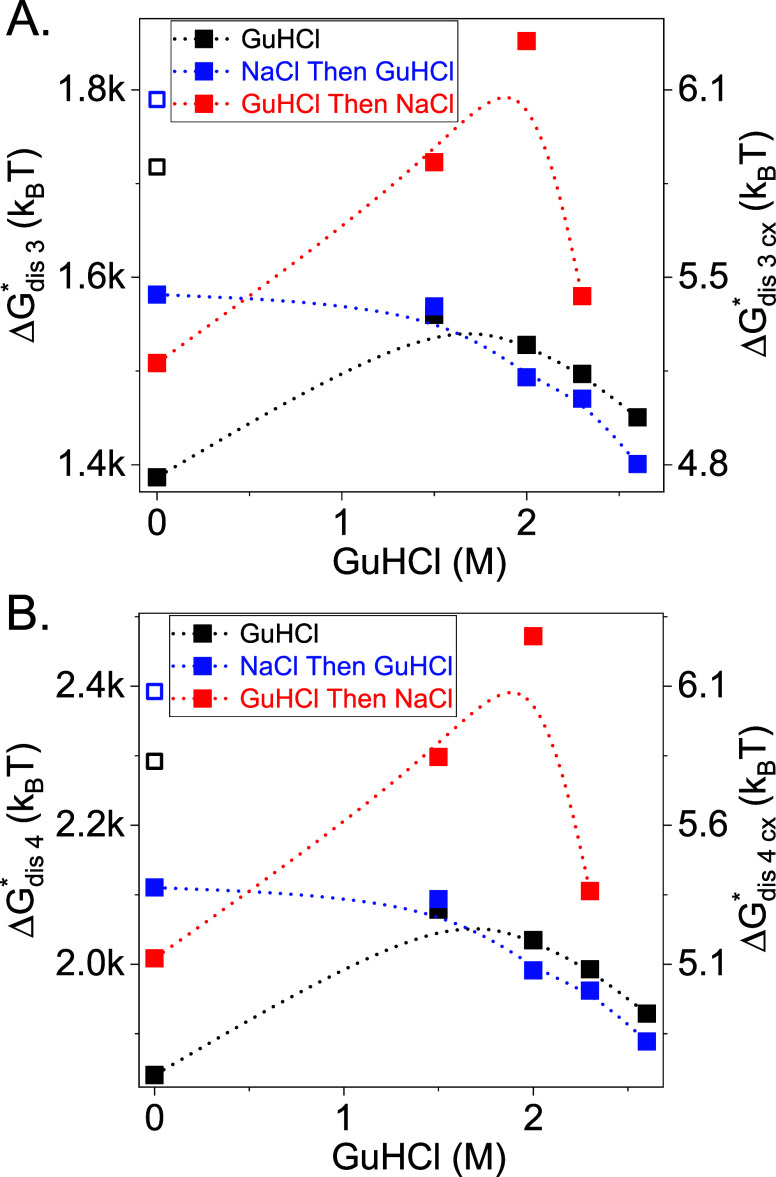
Estimated disassembly energy barriers, Δ*G*_dis,s_^*^ (left vertical axis), or disassembly energy barrier per dimer–dimer contact Δ*G*_dis,s,cx_^*^ (right vertical axis) in the molar fraction scale in units of thermal energy for *T* = 3 (A) and *T* = 4 (B) as a function of GuHCl concentration for the different reactions (solid symbols), as indicated, using eq 8. Open symbols are disassembly barriers in the absence of GuHCl based on the corresponding extrapolated values from Figures 4, their associated mass fractions (see text), and eq 8 in water (open black symbols) or 1 M NaCl (open blue symbols).

Using our data, we also estimated the energy barrier for disassembly on the molar fraction scale by
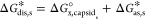
8where  is the total standard Gibbs free energy in the molar fraction scale for the formation of a capsid with *s* subunits (eq 5). The disassembly barriers of *T* = 3 and *T* = 4 capsids were significantly higher than the corresponding assembly barriers (Figure 6), in agreement with an earlier report.^39^ The disassembly barrier for *T* = 4 was higher than that of *T* = 3 but once the values were normalized to the number of dimer–dimer contacts, the barriers per contact were nearly the same (Figure 6), as were the association energies (Figure 4). The addition of a sufficiently high GuHCl concentration decreased the disassembly barrier in all the cases.

In the absence of GuHCl, we distinguish between Δ*G*_cc_^°^(0 GuHCl)_a_ [or Δ*G*_*cx*_^°^(0 GuHCl)_a_] calculated from analysis of the assembly reaction (Figure 4, solid symbols) at zero GuHCl concentration and Δ*G*_cc_^°^(0 GuHCl)_d_ [or Δ*G*_*cx*_^°^(0 GuHCl)_d_] calculated based on extrapolation of GuHCl-induced disassembly reactions to zero GuHCl concentration. Δ*G*_cc_^°^(0 GuHCl)_a_ [or Δ*G*_*cx*_^°^(0 GuHCl)_a_] is in agreement with other published assembly experiments^9,10^ and is consistent with the successful assembly mechanism with minimal kinetic traps.

Below 1.5 M GuHCl, disassembly was undetectable (Figures 3B and 4 blue symbols). At higher GuHCl concentrations, disassembly increased with the GuHCl concentration. The dimer–dimer association standard free energy increased linearly with GuHCl concentration (Figure 4),^60^ letting us estimate the native conformational standard Gibbs free energy by
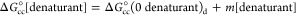
9where *m* is the rate at which the free energy increases with denaturant concentration. m = 0.528 kcal/mol^2^ for reactions where NaCl was gradually added and then GuHCl was added. m = 0.376 kcal/mol^2^ for reactions where GuHCl was added and then 1 M NaCl was added. Δ*G*_cc_^°^(0 GuHCl)_d_ [or Δ*G*_*cx*_^°^(0 GuHCl)_d_] was obtained from a linear fit of Δ*G*_cc_^◦^ of disassembly reactions as a function of GuHCl concentration and extrapolation to 0 M.^16,60^ The extrapolation gave Δ*G*_cc_^°^(0 GuHCl)_d_ = −4.84 kcal/mol in 1 M NaCl and −4.58 kcal/mol in water on the concentration scale or Δ*G*_*cx*_^°^(0 GuHCl)_d_ = −6.07 and −5.81 kcal/mol on the molar fraction scale (Figure 4). These values are 0.75 kcal/mol (in 1 M NaCl) and 1.4 kcal/mol (in water) lower than the corresponding assembly Gibbs standard free energies, Δ*G*_cc_^°^(0 GuHCl)_a_ [or Δ*G*_*cx*_^°^(0 GuHCl)_a_], obtained by direct measurement of the assembly reaction in the absence of GuHCl. Based on mass conservation and eq 5, the extrapolated free energies correspond to mass fractions of 0.014 free dimers, 0.104 *T* = 3, and 0.882 *T* = 4 in water (as opposed to 0.975 dimers, 0.001 *T* = 3, and 0.024 *T* = 4; Figure 2B) and 0.006 free dimers, 0.006 *T* = 3, and 0.988 *T* = 4 in 1 M NaCl (as opposed to 0.064 dimers, 0.025 *T* = 3, and 0.91 *T* = 4; Figure 3B). These data are consistent with hysteresis in the disassembly process, for which a kinetic model was proposed.^16^

The estimated energy barriers for assembly and disassembly and the critical nucleus sizes also exhibited hysteresis. Using the free energies from the extrapolation of disassembly reaction to 0 M GuHCl, Δ*G*_*cx*_^°^(0 GuHCl)_d_ (Figure 4, open symbols) gave lower assembly energy barriers and smaller critical nucleus sizes than obtained from direct assembly reactions in the absence of GuHCl (Figure 5, open symbols). Accordingly, the disassembly energy barriers based on the extrapolated energies were higher (Figure 6, open symbols).

What had been argued was that by removing one dimer from an *N*-dimer capsid, the reassembly of the resulting *N* – 1 intermediate competes with disassembly, creating a kinetic barrier for disassembly. This barrier suggests that the process of disassembly is gradual, in which dimers are taken out one at a time and not by implosion/explosion of the capsid owing to instability.^11^ Furthermore, removing one subunit does not lead to instability because the *N* – 1-mer is the most stable intermediate in the assembly process.

In comparison, pH-driven capsid disassembly of SV40 and CCMV was attributed to increased electrostatic repulsion between capsid proteins weakening the dimer–dimer association free energy.^11,18^ In contrast, GuHCl-driven disassembly of HBV is attributed to weakening of the hydrophobic interactions between capsid proteins and increasing of the barrier for assembly (Figure 5). Even though the denaturation mechanism of GuHCl is unclear,^21^ a plausible mechanism is the hydrophobic planar stacking of GuHCl onto hydrophobic amino acid residues of the protein.^21,24,59^ It is important to note that the Debye screening length was 0.304 nm at 1 M NaCl and decreased to 0.160 nm after adding 2.6 M GuHCl. Even though these changes were small, the increased ionic strength decreased the repulsive contribution of the electrostatic interaction to the dimer–dimer association standard free energy and stabilized the capsid. However, by adding 2.6 M GuHCl, the total association standard free energy increased, suggesting that GuHCl weakened the vdW and/or hydrophobic attraction and destabilized the capsid.

GuHCl, most likely, interacted with buried hydrophobic amino acid residues and weakened the association between the dimers. The preferential interaction of GuHCl with the protein may also provide an additional steric hindrance element, especially if the GuHCl was stacked onto one another and formed clusters.

Disassembly hysteresis has important biological implications; after viruses assemble in the host cell, they must infect other hosts, usually by a passage through harsh environments like air^61^ or the host’s biological defenses.^62−65^ To survive those conditions, disassembly is less favorable. Yet, viruses must disassemble to infect. In vivo, HBV disassembly may be primed by interaction with host proteins^66^ or by the internal pressure of dsDNA of the mature virus compared to the ssRNA of the immature form.^67,68^ In vitro, HBV dissociation is stimulated by low ionic strength and denaturants.

## Conclusions

Understanding the disassembly process of capsids is crucial for understanding the mechanism of viral infection and developing new antiviral drugs.^69^ Disassembly is essential for the viral life cycle as it allows genetic material to be incorporated into infected cells.^17^ Recently, we have shown the disassembly mechanism of SV40 in response to pH increase.^11^ HBV capsid disassembly was initiated by high GuHCl concentrations. To destabilize and disassemble a capsid, GuHCl must increase the dimer–dimer association free energy, Δ*G*_cc_^◦^. Therefore, the amount of disassembly decreased at high NaCl concentration, which decreases Δ*G*_cc_^◦^.^9,10^ At 1 M NaCl, the capsids were stable enough for titration with GuHCl, meaning that the metastable character of the capsids can be tuned with ionic strength. Fitting the titration curves with a three-state model of dimer, *T* = 3 capsid, and *T* = 4 capsid, computed by D+ software,^100^ revealed their mass fractions at each titration point, unraveling the dimer–dimer association free energy. The disassembly and assembly energy barriers and critical nucleus size were also estimated. A hysteresis of capsid disassembly resulted in a detectable energy difference between the dimer–dimer association standard free energy, Δ*G*_cc_^°^(0 GuHCl)_a_ [or Δ*G*_*cx*_^°^(0 GuHCl)_a_], in the absence of GuHCl, obtained from an assembly reaction, and the extrapolated dimer–dimer dissociation standard free energy, Δ*G*_cc_^°^(0 GuHCl)_d_ [or Δ*G*_*cx*_^°^(0 GuHCl)_d_], based on disassembly reactions. Hysteresis was observed also in the estimated disassembly and assembly energy barriers and critical nucleus sizes. Incubation of Cp149 dimers with 2.6 M GuHCl in the absence of NaCl showed that the interactions of GuHCl with the HBV Cp149 dimers are most likely hydrophobic. It seems that the electrostatic screening of GuHCl and the weakening of the hydrophobic interactions, either by steric effects or by increasing the solubility of the hydrophobic surface, led to capsid disassembly. Recent studies of the kinetics of dissociation, at single-molecule resolution, by nanofluidic resistive pulse sensing, suggest two overlapping mechanisms for dissociation.^70^ Capsids remained intact until a threshold of subunits had been removed. In some cases, that threshold was about 25%, consistent with random removal of subunits until a percolation threshold was exceeded.^71,72^ Other particles persisted until 50% of the subunits were removed, suggesting that the particle unraveled while a core of subunits remained intact. In both of these models, free subunits in the solution could reassociate, contributing to hysteresis. An additional contributor to hysteresis is the likelihood of a postassembly conformational transition.^73^

## Accession Codes

The UniProt accession ID of PDB 2G33 is P03147.
